# Ultra-Rapid Categorization of Fourier-Spectrum Equalized Natural Images: Macaques and Humans Perform Similarly

**DOI:** 10.1371/journal.pone.0016453

**Published:** 2011-02-04

**Authors:** Pascal Girard, Roger Koenig-Robert

**Affiliations:** 1 Université de Toulouse, UPS, Centre de Recherche Cerveau et Cognition (CerCo), Toulouse, France; 2 Centre National de la Recherche Scientifique (CNRS), CerCo, Toulouse, France; 3 Institut National de la Santé et de la Recherche Médicale (INSERM), Toulouse, France; Cajal Institute, Consejo Superior de Investigaciones Científicas, Spain

## Abstract

**Background:**

Comparative studies of cognitive processes find similarities between humans and apes but also monkeys. Even high-level processes, like the ability to categorize classes of object from any natural scene under ultra-rapid time constraints, seem to be present in rhesus macaque monkeys (despite a smaller brain and the lack of language and a cultural background). An interesting and still open question concerns the degree to which the same images are treated with the same efficacy by humans and monkeys when a low level cue, the spatial frequency content, is controlled.

**Methodology/Principal Findings:**

We used a set of natural images equalized in Fourier spectrum and asked whether it is still possible to categorize them as containing an animal and at what speed. One rhesus macaque monkey performed a forced-choice saccadic task with a good accuracy (67.5% and 76% for new and familiar images respectively) although performance was lower than with non-equalized images. Importantly, the minimum reaction time was still very fast (100 ms). We compared the performances of human subjects with the same setup and the same set of (new) images. Overall mean performance of humans was also lower than with original images (64% correct) but the minimum reaction time was still short (140 ms).

**Conclusion:**

Performances on individual images (% correct but not reaction times) for both humans and the monkey were significantly correlated suggesting that both species use similar features to perform the task. A similar advantage for full-face images was seen for both species. The results also suggest that local low spatial frequency information could be important, a finding that fits the theory that fast categorization relies on a rapid feedforward magnocellular signal.

## Introduction

The macaque monkey provides one of the closest animal models for studies of the mechanisms of human brain function [1] including cognitive processes such as visual categorization. Recent studies have revealed that monkeys can categorize natural scenes very efficiently (review in [2]). They have shown that Rhesus macaque monkeys are as accurate as humans in categorization tasks involving large sets of images [3]–[5]. Furthermore, these studies also revealed that the categorization can be extremely fast, with behavioural responses reaching a minimum of 100 ms in a forced-choice saccadic task [4]. It is important to stress that such values place severe constraints on the processing involved in such elaborate cognitive tasks. In particular, it is well established that selectivity to complex stimuli is present in the inferotemporal cortex of the macaque (for a recent review, see [6] but neuronal latencies are such that little processing time is available between stimulus onset and a motor output at 100 ms [7]–[9].

One relatively simple hypothesis can be put forward to explain these extremely fast reaction times in cognitive tasks: subjects could perform the categorization on the basis of low-level attributes of the images, putatively processed in lower order areas with faster neuronal responses. Such a hypothesis is supported by the work of Oliva and Torralba who have shown that the gist of a natural scene can be grasped on the basis of the spatial frequency content of the image [10]. In the same vein, in humans, fast saccades are still biased toward images of faces in which phase components are randomized and thus must presumably depend on the 2D amplitude spectrum of the images [11]. Hence, in a categorization task, one cannot formally exclude the possibility that images belonging to one category (animal targets for instance) have a spectral content different from that of images of other categories. This is an important issue since former studies have shown that monkeys can use low-level cues that are unrelated to a category per se, for instance a colour patch, to classify stimuli [12]. One solution to avoid a low-level response bias toward one category consists in normalizing all the images of the study in term of mean luminance and RMS contrast and equalizing them in spectral energy. A recent study [13] showed that human subjects are still able to categorize natural scenes and man-made scenes that have been equalized by giving them the same averaged power spectrum. The main consequences of the equalization process were a slight drop of accuracy and an increase in manual reaction time. Our first aim was to determine whether monkeys can also categorize equalized images and at what speed. In the present study, we successfully trained one rhesus macaque monkey to perform a forced-choice saccadic categorization task of equalized images of animals in natural scenes.

Our second aim was to compare the performance of the monkey with that of human subjects with the same set of equalized images. When tested in the same conditions and with the same images, monkeys are somewhat less accurate but faster than human in a manual go-nogo categorization task of animals in naturalistic scenes [14]. Recent work has emphasised the striking similarity between the cortical representation of categories in both species of primates using passive presentation of numerous natural stimuli [15], [16]. Multidimensional analyses of fMRI in humans and neuronal responses in macaques showed that inferotemporal cortex contains separate representations for animate and inanimate objects in which subcategories like face and bodies are distinguishable. Under the methodological constraint of equalized images, we further explored whether monkeys and humans use the same strategies to categorize the same images in the demanding force-choice saccadic task. We focused on several important characteristics of the images such as the angle of view with which the faces were displayed. Humans are readily able to categorize many different species as animals, even odd-looking ones such as ant-eaters or armadillos. Because humans have an obvious cultural advantage, we examined the similarity of categorization across various types of animals. Both species achieved fast reaction times and have a comparable overall accuracy. They also had a similar accuracy on individual images and gave precedence to full-face and close-up views of the faces of the animal targets.

The last question was related to the theoretical possibility that fast categorization could rely on the quantity of relevant information contained in the low spatial frequencies. Authors have postulated that low spatial frequencies could allow building up a quick hypothesis about the content of the image [17] to help recognition or categorization. In the forced-choice saccadic task, there is little time to elaborate a full description of the image and efficiency could rely on the use of low spatial frequencies. Since images were equalized and the phase was not disrupted, they had all the same global frequency content. However, if the target in the image was more salient because of the combination of local low spatial frequencies, it should have been more easily categorized in the saccadic task, considering the hypothesis of Bar. We investigated the potential role of low spatial frequencies in humans, in a rating psychophysical task.

## Methods

### Ethic statement

All experiments on human subjects were approved by the local ethical committee ‘Comité Consultatif de Protection des Personnes dans la Recherche Biomédicale Toulouse II’ (permit N° ‘Avis N°2-03-34/Avis N°2’). All subjects gave informed written consent to participate in the experiment.

All experiments on the monkey subject were in conformity with the ethical rules of the EEC (EEC, Directive No. 86–609, November 24, 1986). All procedures were in accordance with the Weatherall report, ‘The use of non-human primates in research’ and were fully approved by the local ethical committee named ‘comité regional d'éthique pour l'expérimentation animale de Midi Pyrénées (permit N° MP/04/04/01/05). The surgical procedures necessary for head fixation are described in [4]. No extra surgical procedure was necessary at any time during the present experiment. The general health status of the animal could be monitored every day by competent and authorized personal. The animal was paired-housed during the whole duration of the experiment.

### Behavioural task

One female rhesus macaque monkey (*Macaca mulatta*, age: 13 years, weight: 3 kg) was used in this study. The animal was already expert in the categorization of images by means of saccadic eye movements (monkey M1 in [4]). We used the same behavioural task as in the former study but with a new set of images. The animal sat in front of a screen (Iiyama vision masterpro 512, 75 Hz frame-rate) with the head immobilized (see [4]). Every trial required first the monkey to fixate a central dot (0.15°, 300 to 450 ms fixation period). A gap period with a blank screen (200 ms) followed the fixation dot, then 2 pictures appeared simultaneously (centered on the horizontal meridian at 5 degrees eccentricity, one in each hemifield) with a presentation duration of 400 ms. One image contained an animal (“target”) and the other did not (“distractor”). As soon as the pictures appeared, the monkey was allowed to make a saccade onto the target. Eye movements were monitored by an ISCAN camera (120 Hz). A drop of water was given after each correct trial; errors were indicated by a low white-noise sound and sanctioned by a slightly prolonged inter-trial delay. We kept careful records of the weight of the water-deprived monkey and gave extra water if needed.

Nine human subjects (3 male and 6 female; mean age 26±4 years) were involved in the same categorization task as the monkey in the same experimental setup and room. They were instructed to make a saccade to the picture that contained an animal. The same CORTEX (NIMH CORTEX) program, in a DOS operating system, was used to monitor the behaviour of both the humans and the monkey. The human subjects sat in front of the same screen as the animal, at the same distance (57 cm); the monkey experiments have been terminated 6 months before and the experimental setup cleaned. Human subjects had their head stabilized by a chin and front device. Their eye movements were monitored with the same camera and software as the monkey. On each correct trial, the subjects could hear the sound of the monkey reward system. All subjects gave informed written consent to participate in the experiment.

### Stimuli

**Figure 1:**
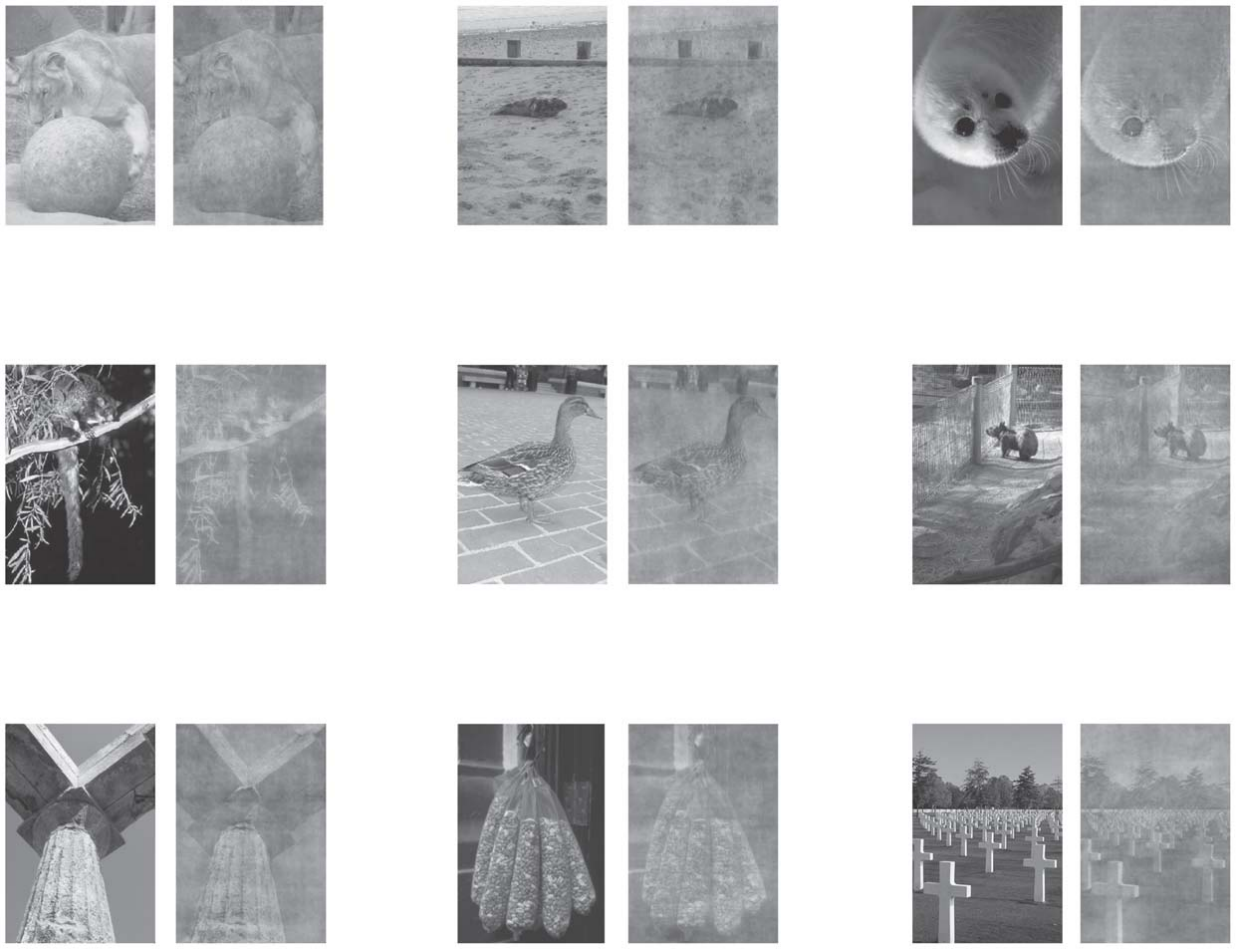
Examples of stimuli. Examples of targets (first 2 rows) and distractors (bottom row) in original grey-level view (left side of each pairs) and equalized version (right side). As illustrated here, distractors often contained salient objects and the target animals could be difficult segregate from the background. In this figure, images appear easier than in the experimental situation since the reader is primed by the original picture.

All images were 8-bit BMP gray level pictures of natural scenes (243×356 pixels, 5×7 degrees of visual angle). About half were taken from the Corel Database and the other half were taken from internet searches in order to display a larger variety of animal species in the targets (see Table S1) and to have a large number of distractors displaying salient objects. All images in the study were first equalized in luminance (mean grey value  = 128) and in RMS contrast (standard deviation of 20.4). In a second step, they were equalized in spectral energy. Equalization was performed by the following operation: we computed the mean power spectrum of the whole set of images (targets+distractors). Then, we applied the mean power spectrum to each image while keeping the original phases [18]. Examples of images before and after the equalization process can be seen in figure 1. The background of the monitor was set to a uniform gray (luminance 14 Cd/m^2^).

In each daily session, the monkey saw 50 pairs of images, 10 of which were composed of completely new images while the remaining ones were familiar. All pairs were displayed in a randomized order and appeared several times in the session. New pairs were composed of pictures that had never been presented to the monkey in either a non-equalized or an equalized version. Although they were repeated along the session, they were considered as new for all trials since they appeared in one session only. Since the monkey performed 51 sessions, it saw 510 different new pairs. Familiar pairs were composed of familiar images taken from a previous study ([4]) in which they were never presented in the equalized version. All the familiar images were present in every session and the pair members were randomly shifted from session to session. Since the animal had not performed the task for several months, we started the experiment with two “warming-up” sessions (two days) in which she performed the task only on familiar non-equalized images (these sessions are not taken into account in the result section). From the third day onwards, only equalized images were presented.

Humans saw only equalized images. In order to draw comparisons, we selected among the 510 new pairs, the 382 pairs that have been presented at least ten times to the macaque, in a given session, and for which we have been able to compute the reaction time offline for each trial (less than 10 trials per pair were available for each of the remaining new pairs and they were not presented to humans). Each human subject saw these pairs, in a random order, in a unique session of 1000 to 1500 trials. Because the humans were not head-fixed as the monkey was, many trials were rejected. The great majority of rejections (15% of the trials) were caused by break in the fixation period. Another 1.6% of the trials were saccades that we rejected offline. We needed 9 human subjects to reach a sufficient number of trials (at least 10 for each pair) for comparison with the monkey.

### Saccadic latencies

We computed saccadic latencies as in [4]. We determined a threshold as the maximum value of the derivative of the horizontal eye trace during the fixation period. The saccadic latency of a given trial was taken as the time between stimulus onset (photodiode signal) and the time at which the derivative crossed the threshold. We then checked that the eye position signal did not return to fixation level for at least five consecutive points. The minimum saccadic reaction time (minimum RT) was defined as the first 10 ms bin of the distribution that contained significantly more correct responses than errors (chi-square test, p<0.05). This bin had to be followed by 5 consecutive bins reaching the same criterion.

### Role of low spatial frequencies

We sought to link the performance obtained in the saccadic task to the low spatial frequency content of the images. All 382 pairs of equalized images that were used in humans were low-pass filtered (2DGaussian with a cut-off frequency of 6 cycles/image [19]. Each target randomly appeared left or right of the midline and each pair of images was displayed during unlimited time. We asked 5 naïve human subjects to rate the presence of an animal in each pair by entering on a keyboard two answers (left/right presence and rating from ‘just guessing’ to ‘clearly seen’ on a 1 to 5 scale). Images that were rated 5 were considered by the subjects as clearly containing an animal whereas a rating of 1 meant they were just guessing. In the analysis of the data, a rating index was computed by multiplying the ratings by −1 if the subject localized an animal in the distractor image or multiplying by 1 in case of correct response; hence, as there were 5 subjects, the rating index could vary form −5 to 5 excepting 0. There were no time constraints and no head restraint in this task. The subjects were in the same age range as the subjects that participated in the saccadic task. They had normal or corrected to normal vision and had not seen the images before.

## Results

### Monkey performance accuracy

**Figure 2:**
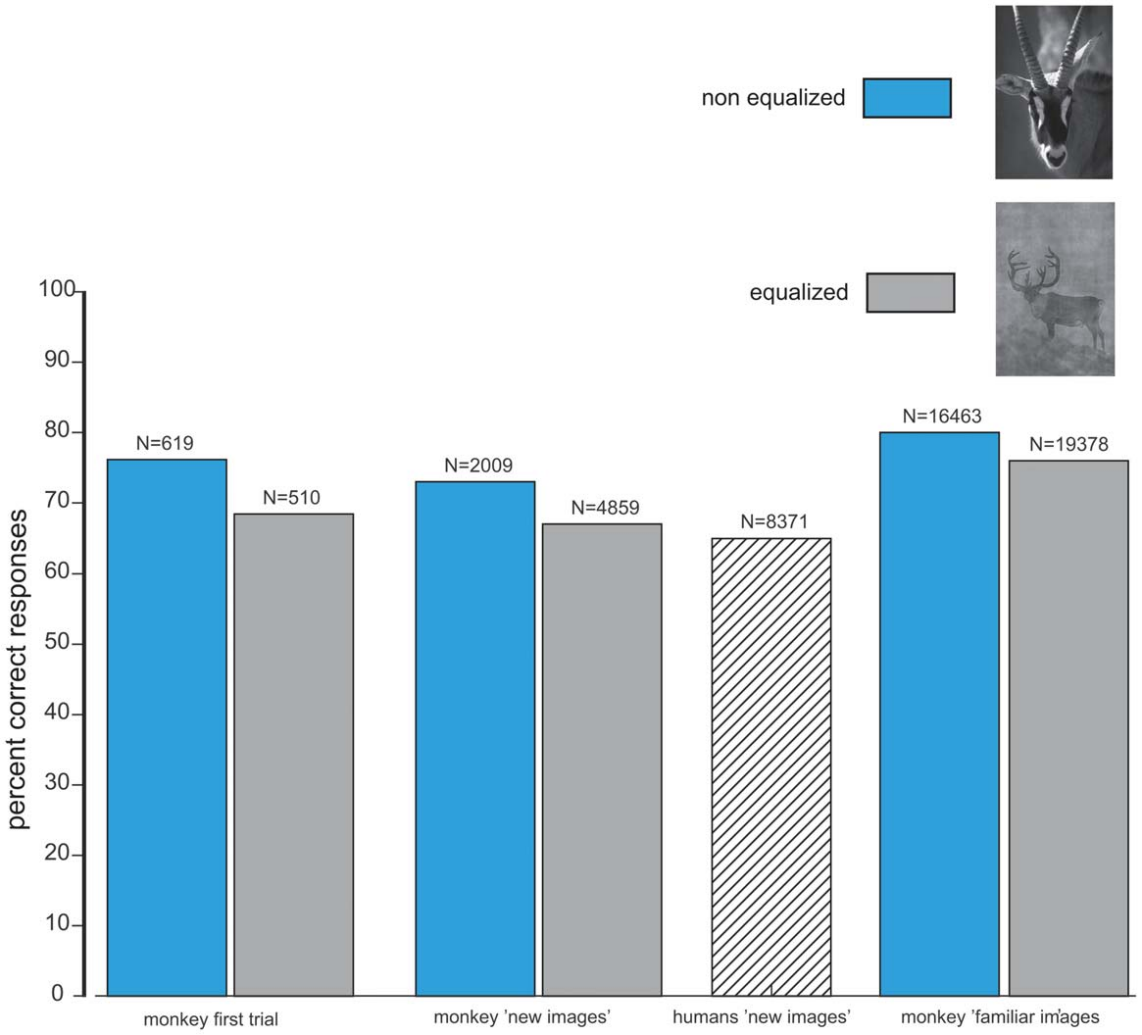
Performance accuracy. Bar plot of the percent correct responses obtained by humans and the monkey on equalized (grey) or non-equalized images (blue, former study). The number of trials is indicated on top of each bar.

**Figure 3:**
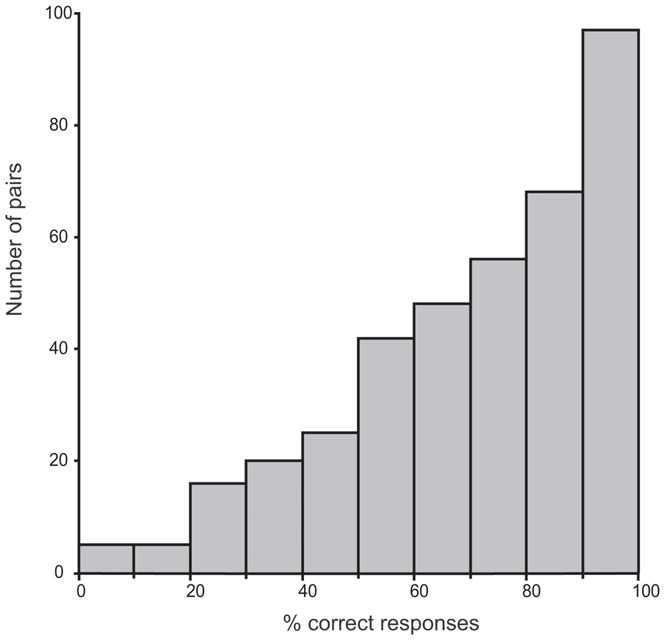
Accuracy on individual pairs of images. Distribution of the percentage of correct responses of the monkey with individual new pairs of stimuli that were presented for at least 10 trials.

The monkey performed the task remarkably well. The overall score of the animal, based on 24237 saccades, was 74.35% correct (figure 2). However, as expected given the degraded aspect of the images, the performance was lower than she had achieved previously with non-equalized images (79.3%, χ2 = 125.35, df = 1, P<0.0001). In order to rule out any rote learning strategy that would have allowed the monkey to solve the task by memorizing stimulus/reward association, we assessed the ability of the monkey to categorize equalized images in the 510 new pairs. The overall mean score of all trials with new images (n = 4859) was 67.48% correct responses, which is significantly above chance (χ2 = 306.43, df = 1, P<0.0001) but below the performance obtained with non-equalized new pairs in the former study (χ2 = 22.48, df = 1, P<0.0001). The performance was good across the different images since among the 510 new pairs, the monkey performed above 50% correct for 371 pairs and 90 pairs elicited 100% correct responses. Even more important is the response to the very first trial on which a given pair appears, since in that case we are absolutely sure that the monkey could not respond on the basis of a simple stimulus-reward association. The mean percentage of correct responses for the very first occurrence of each of the 510 pairs of new images was 68.43% and clearly above chance level (χ2 = 35.87, df = 1, P<0.0001). If we restrict the analysis to the 382 pairs that were presented at least 10 times, the overall score was 67.25% correct (4025 trials) and above chance level (χ2 = 247, df = 1, P<0.0001). The median accuracy on the different pairs was 70%, 97 pairs gave above or equal to 90% correct responses, 52 pairs gave 100% correct responses, and only 5 pairs were systematically miscategorised (figure 3).

The monkey made significantly more correct responses to familiar images than to new images (76.08% correct, χ2 = 150.62, df = 1, p<0.00001). This performance was significantly below the score of 80% obtained in the previous experiment on familiar pictures (χ2 = 82.06, df = 1, p<0.0001). Interestingly, responses to familiar images were rapidly better than those to new images: the performance of the first occurrence of the 40 familiar targets was 78% correct on the first session where they appeared. The median accuracy on different familiar images was 78.5%. Despite this overall high level of accuracy, the monkey did not exceed chance level on 3 familiar images. These 3 targets depicted respectively a panther, a lemur and a giraffe. This strengthens the view that the performance of the saccadic task did not depend on rote learning: even these 3 images were familiar ones, they were paired with a different distractor at each session and the monkey could not learn them.

### Human performance accuracy

Each human subject took part in one experimental session only. They each saw the 382 new pairs that have been presented at least 10 times in the monkey. Each subject performed between 1000 and 1500 trials of which a substantial proportion (15% on a total of 10080) were aborted or rejected (1.6%) since it was not possible to keep the subjects' head as still as in the head-fixed monkey's experiment. However, we decided that the human subjects should not participate in more than one session to avoid a familiarisation with the images. As a consequence, the overall score was based on 8371 saccades. The overall level of accuracy of the human subjects was 63.74%. All subjects performed above chance level with the worst one reaching 54.64% and the best one 79.84% correct responses. The performance is substantially less than the 90% correct reported with non-equalized images [20]. Figure 2 shows the accuracy for both the monkey and the human subjects for the present and the former study.

### Saccadic latencies

**Figure 4:**
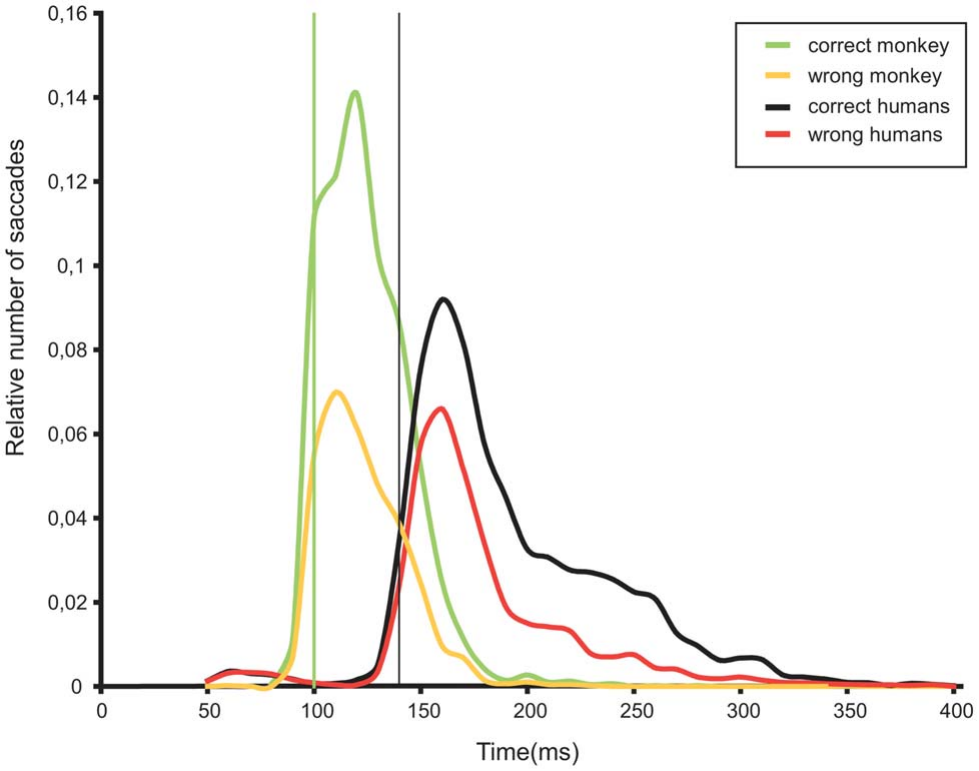
Distribution of correct and incorrect saccades (relative number of trials) for humans and the monkey. Vertical bars indicate the minimum reaction times (for monkey in green and humans in black).

Figure 4 shows the distribution of saccades latencies obtained for the 382 equalized new pairs that were common to the humans and the monkey. The monkey performed the task very quickly: the median reaction time for correct trials was 121 ms and the minimum reaction time was 100 ms (latency range 95–104 ms). If we consider all 510 pairs of new images used in the monkey, median and minimum reaction time were not different and the distribution of saccades latencies is very similar to the one shown in figure 4 (not shown). Familiar images (monkey only) also lead to a median reaction time of 121 ms but a slightly shorter minimum reaction time (90 ms, not shown).

Correct trials in humans displayed longer latencies than correct trials in the monkey (Mann–Whitney, *U* = 1.34×10^7^, n1 = 2707, n2 = 5336, *P*<0.0001). The median human reaction time (correct trials) was 172 ms and the minimum reaction time was 140 ms (range 135–144 ms). The median reaction time was considerably shorter than the 228 ms reported by Kirchner and Thorpe ([20]). Individual median reaction times (correct trials) ranged between 159 and 197 ms except for one subject at 255 ms.

### Inter-species comparisons

This second part of our study was intended to explore the similarities between humans and monkey by making extensive comparisons on the 382 common individual target images.

The examination of the human saccadic distribution in figure 4 (and saccadic distributions of individual subjects) suggests that reaction times below the 120 ms are likely to be anticipatory saccades. Indeed, the overall performance for saccades below 120 ms is 40% correct only. Such anticipatory saccades were virtually absent in the monkey distribution (only 3 latencies were below 80 ms). Hence, for comparison with the monkey, we kept human latencies that were between 120 ms and 400 ms (7907 saccades, 65% correct) and monkey latencies between 80 ms and 400 ms (4022 saccades, 67.25% correct). On this set of data, the performances of both species were quite similar, although the monkey was statistically slightly more accurate (χ2 = 6.35, df = 1, p = 0.0118).

**Figure 5:**
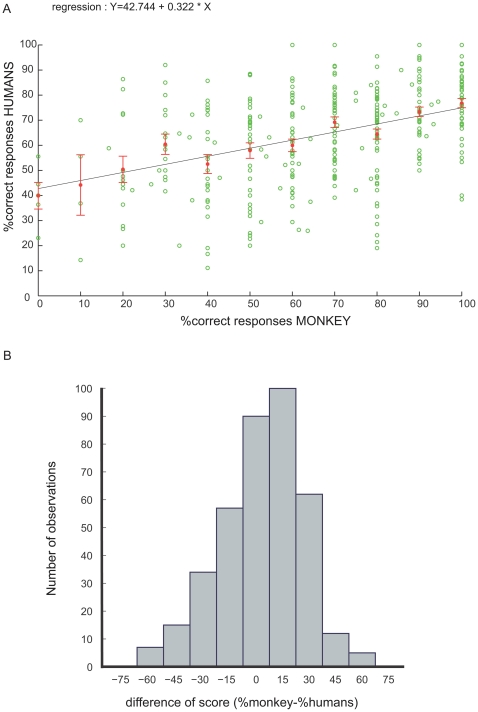
Comparison of accuracy of humans and monkey. 5a: regression plot of the percentage of correct responses for humans as a function of the score of the monkey for each individual pair of stimuli. Red dots indicate the mean score of humans for each 10% correct bins. Error bars show the standard error of the mean. 5b: histogram of the difference in performance of the monkey and the humans on individual pairs of images.

An important issue was to test whether both primate species use a similar strategy by looking at the performance on the same pairs of images. The relationship between the performance of the humans and the monkey on individual images is shown with the linear regression plot in Figure 5A. The regression equation (Y = 42.744 + 0.322 * X; R^2^ = 0.177) indicates that there was a slight tendency for humans and the monkey to perform similarly on each pair of images. Another way to express the similarity of performance between both species is by using the distribution of the difference of percent correct responses (Figure 5b). The distribution is approximately centred on zero and shows that most targets did not elicit more than 10–20% difference of performance between the monkey and humans (the median of the distribution is at 5% difference). However, since the distribution is quite broad, there were a number of images on which the monkey and the humans performed differently.

**Figure 6:**
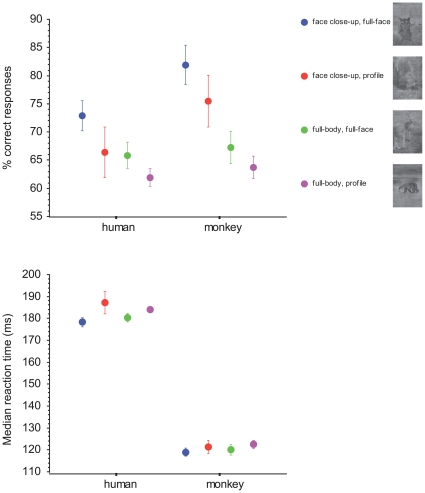
Advantage for faces. Percentage of correct responses and median reaction times of the monkey and human subjects to different views of the face in the target stimuli (mammals).

We further explored which characteristics of the images could lead to similar responses in both species. We only focused on the content of the targets and not on that of the distractors. The first characteristic we examined was the presence of faces, which are potentially attracting features in the targets. To compare human and monkey performances, we split the images into several subcategories according to the status of the face. Each target could be either a close-up view of a face or a full-body presentation; these two kinds of targets were further split in two groups according to the orientation of the face (full-face view or profile view). Only targets belonging to the class of mammals were included in this analysis and 15 images were excluded because they contained several individuals with different head orientations; hence the analysis relied on 268 images. The monkey and the humans had a similar response profile with respect to faces (figure 6). Face close-up views elicited better responses than full-bodies, this being particularly prominent in the monkey (U Mann-Whitney; monkey: U = 3664, p<0.0001; humans: U = 4565, p = 0.0079. Median latencies were slightly shorter for face close-up than for full-bodies, but only in the monkey (monkey: U = 4908, p = 0.046; humans: U = 5715, p = 0.67). Furthermore full-face presentations elicited better scores than profile views (Monkey: U = 6933, p = 0.013, humans: U = 6934, p = 0.013). Full-face presentations elicited shorter median latencies than profile views in the monkey (Monkey: U = 7107, p = 0.03, humans: U = 7365, p = 0.074). Within close-up and full body categories (figure 6), the performance for full-face was always above that for profile views but this did not reach statistical significance in both monkey and humans. Finally, in terms of minimum latencies, face close-up views were better for the monkey (90 ms) than other views (100 ms) whereas in humans, all kind of views elicited a 140 ms minimum reaction time except profile views (170 ms). In summary, full-face or face close-up views were the most efficient stimuli both in terms of accuracy and speed.

**Figure 7:**
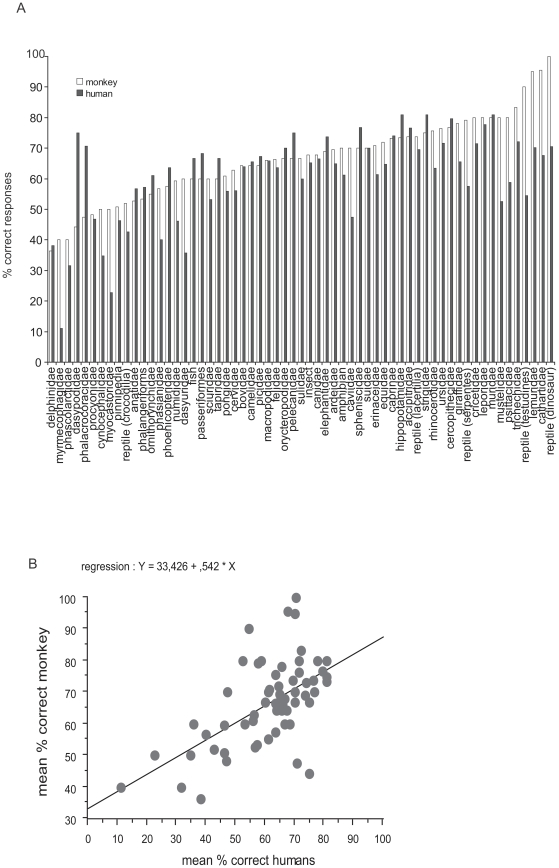
Responses to different animal families. 7a: bar plots of the respective percentage of correct responses for humans and for the monkey to different animal families. The number of stimuli contained in each family is very variable. 7b: mean percent correct responses of the monkey to different families of animals plotted against corresponding responses of human subjects. Also shown is the regression line.

A potential difference between the monkey and the humans is that the latter will have already seen exemplars of many species on different media, something that is much less likely for our monkey, who was born in captivity. Hence, we examined if both the monkey and humans respond similarly to the different families of animals depicted in the targets. The term family here corresponds in most cases to the appropriate taxonomic family (Table S1) for mammals and birds but corresponds to the class for insects and fish, and the order in the case of reptiles. Note that although we chose a wide spectrum of animal prototypes, the experiment was not designed to present an even number of targets in each family. Figure 7a shows the respective performances of the monkey and humans on the different families of animals (corresponding to the 382 pairs in common). The monkey performed above 50% correct for most families. In most cases, the monkey was close to and even better than humans. The monkey was successful in categorizing some of the oddest animals (according to human standards) like the hedgehog (erinaceidae, 71% correct), the aardvark (orycteropodidae, 66.6% correct). She had difficulties for armadillos (dasypodidae, 44% correct) that differ from many species by having a very odd texture that was still visible in the equalized pictures. Both humans and monkey had difficulties with myrmecophagidae (anteaters) and procyonidae (coatis, raccoons). Figure 7b shows that the mean percent correct responses obtained by humans and the monkey on the different families are positively and significantly correlated (regression equation: y = 33.426 + 0.542 * x; R^2^ = .342, P<0.0001).

The overall good performance of the monkey on various families could potentially result from the fact that, among the different species of target animals, some were also used (although on different images) in the familiar images that were repeated across sessions (for instance, the bald eagle was one of the familiar images and 5 other new pictures contained a bald eagle (see Table S1) This concerned 92 images out of the 382 targets. The monkey performed better on the images containing a familiar species (overall performance 72% correct) than on those depicting an unfamiliar one (overall performance 66% correct). This difference was significant (χ2 = 12.4, df = 1, P<0.0005). This was also the case if we consider only the very first presentation of each image (75% correct for familiar species and 64% for new species). Interestingly, humans, who never saw the familiar images of the monkey and had only one session, performed similarly on both groups of images (overall respective performances were 66% and 64.5% correct; χ2 = 1.56, df = 1, P = 0.21). Hence, in the monkey, the advantage for familiar images generalized to other exemplars of familiar species. Could familiarity generalize to similar animal species that were not strictly the same? For instance, if a familiar image depicted a leopard, could the monkey give better responses to cats or tigers that were not represented in the familiar pictures? These ‘close-to-familiar’ species involved 103 images (excluding the former 92 with familiar species in the strict sense). The monkey performed better on this subset of images (68.4%) than on non-familiar images (64.4%) but the significance was much lower (χ2 = 5.11, P = 0.0239). Humans, like the monkey, performed better on the subset of ‘close-to-familiar images’ (66.6% and 63.4% respectively, χ2 = 6.51, P = 0.01). For both the monkey and humans, there was no difference in term of speed for familiar versus non-familiar species (monkey: U = 1962441, P = 0.0927; humans: U = 5556532, p = 0.2078). For the monkey, the median reaction time was 120 ms for both familiar and non-familiar species and the minimum reaction time was 100 ms in both cases.

### Role of low spatial frequencies

**Figure 8:**
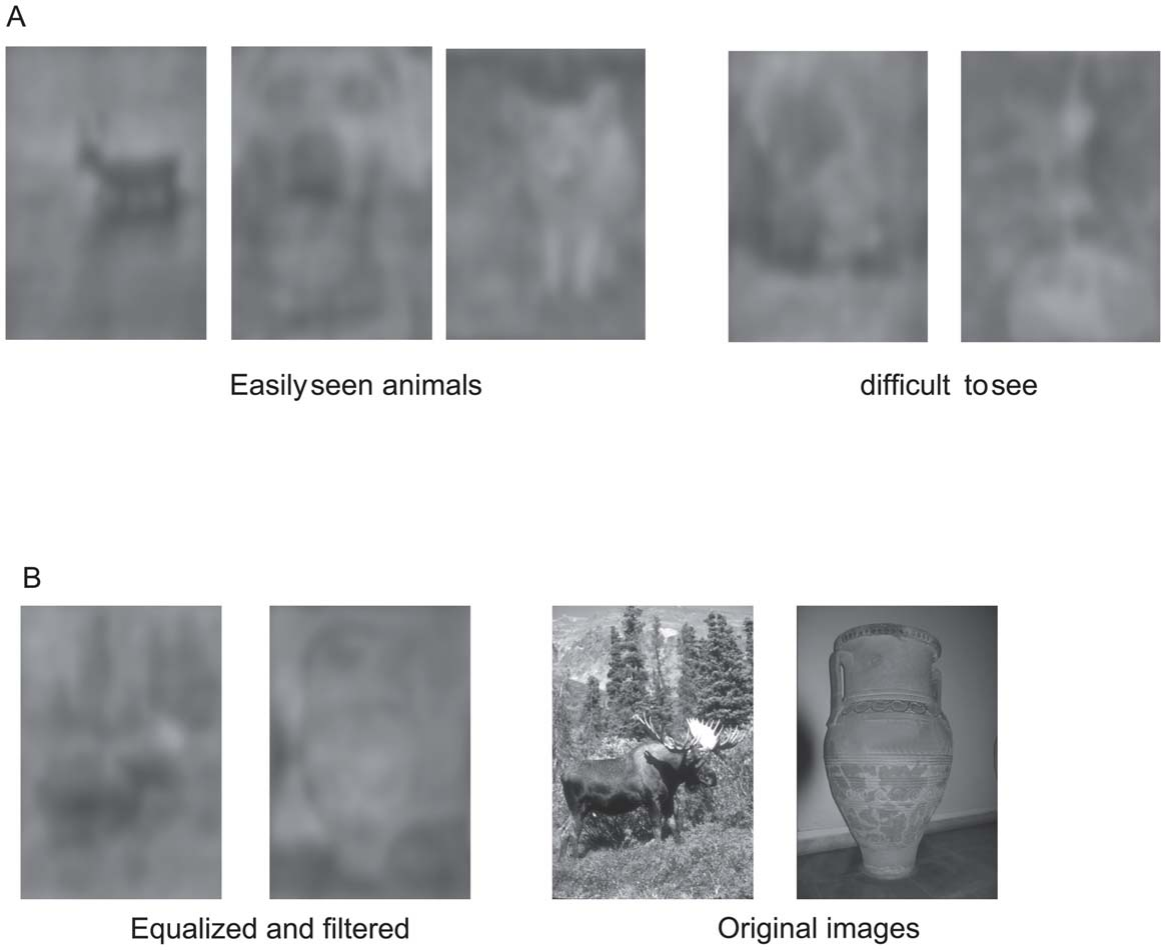
Examples of low pass-filtered equalized images. 8a: targets with various levels of visibility (3 left images are easily visible, 2 rightmost images are difficult). 8b: example of a pair that was miscategorised by both the humans and the monkey. The equalized and filtered version is shown on the left side and the original images on the right.

**Figure 9:**
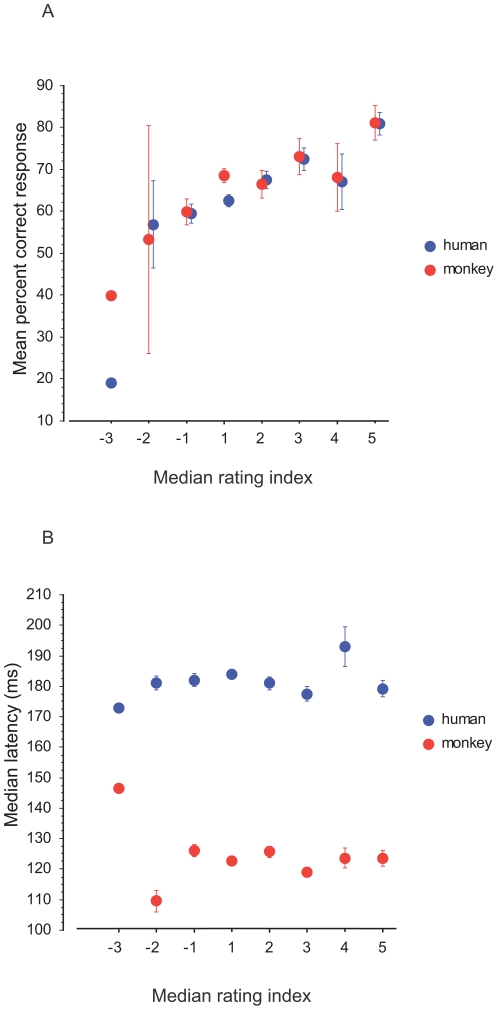
Role of low spatial frequencies. Scores obtained by humans and monkey in the saccadic experiment are plotted as a function of the median ratings given by different human subjects. Ratings ranged from 1 to 5 and were multiplied −1 for wrong responses.

In this experiment, the low-pass filtered images had an extremely degraded visual aspect. However for some of them, one can clearly detect the animal in the picture (figure 8a). The five new human subjects (who did not take part in the experiment with saccades) managed to correctly locate the animal in 75.76% of the pairs. The performance increased with confidence in ratings. Humans rated 60% of the trials as 1 (example of targets from those trials in fig. 8a right). Although 1 meant pure guessing, they performed above chance (67.73% correct, χ2 = 74.87, df = 1, P<0.0001) in that case. When they were sure of their choices (rating 5, 9% of the trials, figure 8a left), they reached 96% correct responses. For intermediate ratings (2, 3 and 4), the respective performances were 83.28, 89.63 and 89.67% correct. It is then interesting to compare the score of the forced-choice detection of the 5 subjects with low-pass filtered-images with the performance obtained in forced-choice saccadic detection by the monkey and the previous human subjects. For each trial made by a subject, we computed the rating index such that the rating given for each pair of images is multiplied by −1 if the response is incorrect and by 1 if correct. Figure 9a shows the mean percent correct responses of the humans and the monkey to individual pairs in the saccadic task as a function of the median rating index obtained by the 5 human subjects on the same filtered pairs. The example in figure 8b illustrates an extreme case of a pair with a median rating index of −3 and that led to 19% and 40% correct responses in humans and monkey respectively in the saccadic task. The data showed a similar trend for the humans and the monkey: the best performance in the saccadic task is obtained for those pairs that had the higher rating index. We did not observe a correlation of the median rates with median latencies for both species (figure 9b).

## Discussion

### Main results

These results confirm the ability of primates to perform this high-level task by means of saccadic responses [4], [20]. The main result of the present paper is that even with images equalized in Fourier-spectrum, both monkeys and humans can efficiently perform the ultra-rapid categorization of animals in natural scenes. Despite the degraded visual aspect of the stimuli, each subject performed above chance level. Importantly, the monkey also readily performed well above chance on equalized images. The monkey made accurate discriminations on images that were completely new, and even on the very first presentation (68.5% correct). The performance on first trials is an important issue considering the work of Cook and Fagot [21] who found that baboons can form long-term memory traces of numerous images from the first trial of presentation. Note here that the degraded aspect of the images is such that the performance with new images is below usual acquisition criteria for discrimination learning (for instance 75% correct in [12]) but mean accuracy on new images was above chance and comparable (if not better) than human subjects.

The use of equalized images rules out the possibility that target animals could be discriminated solely on the basis of a bias in the global statistics of contrast, luminance or spatial frequency content as suggested by computational studies [22]. However, we cannot make the suggestion that the amplitude spectrum has no contribution to the task. Indeed, the most noticeable effect of equalization was a moderate reduction in performance in comparison to the results obtained with ‘intact’ images'. Reductions of performances have also been reported by other authors who recently assessed in humans the role of the amplitude spectrum and its interaction with the phase content in similar tasks [23], [24]. In our study, this reduction was similar in humans and the monkey since both species reached an overall level of accuracy around 65%–68%. In the monkey, the decrease from 73 to 68% (for new images) is in the same range as the decrease of 6% observed in monkeys that performed an animal categorization task when the luminance is altered [25]. Joubert and collaborators [18] also found a decrease of 6% in human accuracy with equalized images in a go/no-go task but on a different category discrimination (natural vs. man-made “context”). However, the mean accuracy of our human subjects was much lower (63.74%) than in Kirchner and Thorpe's experiments (90%) that also used saccadic responses. This drop in accuracy was in the range of the 16% drop in accuracy observed by Wichmann and collaborators when their human subjects performed a saccadic categorization of animals on a subset of images in which both the target and the distractor were ‘difficult’[24]. These authors suggested that this difficulty might be a consequence of how photographers adjust the depth-of-field: difficult animals were not segregated from the cluttered background whereas difficult non-animals were segregated from a blurred background (hence being confusable with a portrait of a living subject). We think that the relatively low mean scores of our subjects comes from the fact that we have chosen our images with a bias towards difficulty and the use of distractor stimuli that nearly always contained a salient object. The results of the rating experiment suggest that it was the case. Let us consider the case of a pair of stimuli such that an animal was very well segregated and the distractor a uniform desert scene: as low pass filtering would not strongly affect the appearance of the target, subjects would have given a rate of 5. However, this was rarely the case (for instance we took care to select distractors with salient objects rather than with uniform landscapes) and the subjects actually reported that they were guessing for 60% of the low-pass filtered pairs, a result that argues strongly that our image set was particularly biased in favour of difficult image pairs.

### Mechanisms of the categorization task

In our task, subjects are under time constraints that would encourage the use of processing strategies that could have been inherited from a common ancestor [2]. One possibility for an efficient categorization is a coarse holistic analysis of objects based on fast processing of low spatial frequencies [7], [17], [26]. The model of Bar [17] is an interesting framework which postulates that a coarse (low spatial frequencies) global magnocellular afferent information rapidly reaches the orbitofrontal cortex. This region then sets up predictions about what the stimulus was and sends back possible matches to be validated in ventral regions including inferotemporal cortex. The advantage is a reduction in the number of possible solutions to make recognition more efficient. Let us examine whether our data are consistent with this framework:

In the rating task, the human subjects saw a low-pass filtered version of the images and could correctly detect the animal in the majority of cases. Performance was correlated with the percentage of correct responses obtained with saccadic responses to the non-filtered versions of the images. This means that images were correctly categorized more often when they were easily understandable in their low-pass filtered version. Furthermore, it should be recalled that in the saccadic task, the images were centred at 5° of eccentricity, where low spatial frequency processing is even more important than in the rating task, which used free viewing. Interestingly, the performance of our human subjects with equalized images compares in terms of percent correct responses with that obtained by other authors with images below 10% contrast, a condition in which discriminability was reduced [25]. Performance was also close values obtained when categorization is done in the far peripheral visual field [27] where the influence of the magnocellular pathway is dominant.

More evidence about a predominant contribution of the magnocellular pathway to the categorization task comes from the reaction times of the subjects. Both species performed the task with very fast reaction times that were in the range of the latencies reported for non-equalized images. Minimum reaction times for both the monkey and the humans were virtually unchanged with respect to previous studies [4], [20]. However median reaction times decreased in particular with human subjects (56 ms shorter). This could result from the fact that we used 400 ms presentation time instead of 20 ms in Kirchner and Thorpe's study. Indeed, recent studies [11], [28] used 400 ms presentation time in a face detection task and obtained mean median reaction times, in humans, of about 180 ms, roughly equivalent to the median reaction times of 172 ms seen with our subjects. The extreme rapidity of the saccades places strong constraints on the brain mechanisms underlying the processing of complex stimuli. The minimum saccadic reaction times around 100 ms and the distribution of neuronal latencies in different cortical areas [7], [29] preclude the possibility that the categorization process uses multiple iterations between brain regions before the motor response. There is indeed converging evidence from different experimental techniques that visual information rapidly reaches the cortical frontal regions. Brain recordings in patients have demonstrated very short latencies in the frontal eye fields [30] reaching the amazing value of 45 ms with depth electrodes [31]. MEG and FMRI experiments in humans show that in a picture recognition task, the orbitofrontal cortex is rapidly activated by visual signals carrying low spatial frequencies (the reported MEG activity starts to develop before 100 ms), that could well originate from fast dorsal magnocellular pathways [32]. Our results point to a fast recognition mechanism based on low frequency contents that fits with Bar's framework, although our use of equalized scenes makes it unlikely that a simple categorization rule could be used. Intermediate level cues such as specific contours [33]–[35], may reflect the set of templates elaborated in frontal regions (after the arrival of the fast feedforward magnocellular information) in order to generate a set of initial hypotheses.

Have we some evidence of such ‘templates’ from our data? Faces are known to have a special significance and attractiveness in primates [36]–[38]. Recent fMRI investigations in both macaques and humans revealed that more brain areas are devoted in face processing than in other body parts [39]. Furthermore, fMRI reveals that face patches have the same relative size in the cortices of humans and monkey [40]. In agreement with these studies, we found in both the monkey and humans a similar trend towards much higher performance with full-face and close-up views of faces with respect to profile and full-body views. Although there is clear evidence of a special status of conspecific faces through expertise [41]–[43], we found that close-up view of full-faces of a large variety of animals (at least mammals) were also more attractive than profile views and full bodies. Hence prototypes of faces are the most obvious candidates as default templates used for guessing the identity of the input stimulus in fast categorization. Our results fit with the recent results of Crouzet and collaborators [28] who found an excellent saccadic detection of conspecific faces in humans and of Fletcher Watson et al [44] who found systematic attraction by faces in free-gazing of natural scenes. Reactions are extremely fast in all these studies. However, more experiments are required to decide between two alternatives. The first one would rely exclusively on feedforward mechanisms, with no time for the use of feedback. The second possibility is that a fast top-down signal carrying the most probable guess (the face) comes into play to trigger fast reaction times. However in that case, because of the saccadic response constraint, it would have no time to be compared with the slower detailed information arriving into the ventral pathway to validate the guess (latencies were not longer for difficult pairs and this was also the case in the study by Wichmann et al. [24]).

### Role of familiarity

In addition, our results seem to indicate that the formation of templates can be quite rapidly modulated ‘on line’ over the period of the experiment sessions. Humans seem to be perfectly able to categorize even very unusual animal species as animals (at least for vertebrates). Each one of us is able to recognize a platypus or an anteater as an animal, and we can even do the same thing for very unusual prehistoric or even imaginary species [45]. The contribution of innate or cultural factors to picture recognition may not be a trivial issue but some studies have shown that people remote from the imaged-overloaded ‘modern’ civilization can recognize the presence of animals in pictures [46]. Since macaque monkeys do not normally have access to human media (although they could see some occasional TV programs as enrichment in our animal facility), it was interesting to assess the monkey's cognitive capacities in categorizing diverse types of animals. As a whole, the monkey performed similarly to humans for a large variety of families of mammals and members of other animal classes. Nevertheless, the correlation of the performances of both species on individual pairs of stimuli was rather modest. It is then important to emphasize the fact that the monkey made correct responses even if the animal targets belonged to a species that had never been presented before, although she performed more accurately on images that contained a species already presented in familiar images. In contrast, humans do not get advantage from their cultural background in the task since the percentage of correct responses obtained by humans and monkey on the non-familiar images are very similar (humans perform below the level reached by the monkey with familiar images). Hence the higher scores obtained with familiar species by the monkey is likely to be caused by a priming by the familiar images that were randomly interleaved with new ones rather than a natural propensity of monkeys to recognize these species or an inadvertent bias towards ‘easy features’ contained in these images. The effect of familiarity seems to occur rapidly since responses to first presentation of familiar species are about 10% better than responses to first presentations of new species. Determining to what extent this familiarity process takes place in the frontal cortical regions would be an interesting future experiment since, in the framework of Bar's model, this may influence the selection of the templates that are used to perform the task.

## Supporting Information

Table S1 **Details of animal species used in the study.** Each line corresponds to one target. Columns indicate the class, the family, the common name and the scientific name. Last two columns indicate whether the image was used in humans and whether the animal target belonged to the familiar species seen by the monkey. At least 165 species were used. 97 could be determined with certainty; the others were undetermined and could belong to more than 68 different species. For mammals and birds, these species belonged to 56 different taxonomic families (53 used in both monkey and humans), with the following deliberate misclassifications: passeriforms (order), caprinae (subfamilia), phalangeriforms (suborder) for possums, echidna and okapi were deliberately misclassified in erinaceidae and equidae respectively, according to their aspect. Reptiles, amphibians, insects and fish were considered under the class name only.
(XLS)
